# Implementing measurement-based care (iMBC) for depression in community mental health: a dynamic cluster randomized trial study protocol

**DOI:** 10.1186/s13012-015-0313-2

**Published:** 2015-09-07

**Authors:** Cara C. Lewis, Kelli Scott, C. Nathan Marti, Brigid R. Marriott, Kurt Kroenke, John W. Putz, Peter Mendel, David Rutkowski

**Affiliations:** Department of Psychological and Brain Sciences, Indiana University, 1101 E. 10th St, Bloomington, IN 47405 USA; Department of Psychiatry and Behavioral Sciences, University of Washington, School of Medicine, Harborview Medical Center, School of Medicine, University of Washington, 325 9th Ave, Box 359911, Seattle, WA 98104 USA; Abacist Analytics, PO Box 11581, Austin, TX 78711 USA; Regenstrief Institute for Health Care, RG-6, 1050 Wishard Blvd., Indianapolis, IN 46202 USA; Centerstone Research Institute, 645 S. Rogers Street, Bloomington, IN 47403 USA; 1776 Main Street Santa Monica, Box 359911, California, 90401 USA; W.W. Wright Education Building Indiana University, Bloomington, IN 47405 USA

## Abstract

**Background:**

Measurement-based care is an evidence-based practice for depression that efficiently identifies treatment non-responders and those who might otherwise deteriorate [1]. However, measurement-based care is underutilized in community mental health with data suggesting fewer than 20 % of behavioral health providers using this practice to inform treatment. It remains unclear whether standardized or tailored approaches to implementation are needed to optimize measurement-based care fidelity and penetration. Moreover, there is some suggestion that prospectively tailored interventions that are designed to fit the dynamic context may optimize public health impact, though no randomized trials have yet tested this notion [2]. This study will address the following three aims: (1) To compare the effect of standardized versus tailored MBC implementation on clinician-level and client-level outcomes; (2) To identify contextual mediators of MBC fidelity; and (3) To explore the impact of MBC fidelity on client outcomes.

**Methods/design:**

This study is a dynamic cluster randomized trial of standardized versus tailored measurement-based care implementation in Centerstone, the largest provider of community-based mental health services in the USA. This prospective, mixed methods implementation-effectiveness hybrid design allows for evaluation of the two conditions on both clinician-level (e.g., MBC fidelity) and client-level (depression symptom change) outcomes. Central to this investigation is the focus on identifying contextual factors (e.g., attitudes, resources, process, etc.) that mediate MBC fidelity and optimize client outcomes.

**Discussion:**

This study will contribute generalizable and practical strategies for implementing systematic symptom monitoring to inform and enhance behavioral healthcare.

**Trial registration:**

Clinicaltrials.gov NCT02266134.

**Electronic supplementary material:**

The online version of this article (doi:10.1186/s13012-015-0313-2) contains supplementary material, which is available to authorized users.

## Background

Depression is one of the world’s leading causes of illness burden [3], costing the United States alone over $80 billion annually due to lost work and wages [4]. There are now many evidence-based practices (EBPs) for the treatment of depression, but these practices remain largely unavailable to clients receiving services in community mental health centers (CMHCs). One reason for this paucity of EBPs is that CMHCs have few resources available for the intensive training plus consultation opportunities that appear necessary to retrain therapists in complex EBPs such as Cognitive Behavioral Therapy (CBT; e.g., [5–7]). Moreover, CMHC therapists bring unique values, attitudes, and skill sets that often misalign with EBPs such as CBT (e.g., [8, 9]). The traditional approach to improving client care—integrating a complex, multi-component EBP like CBT into care delivery—is faced with significant challenges.

Conversely, measurement-based care (MBC) is a relatively simple evidence-based intervention framework [10]. MBC, by definition, is the practice of using routine symptom measurement to inform treatment [11]. MBC is an evidence-based framework that has established effectiveness, broad reach, and multifaceted utility for enhancing usual care. In terms of effectiveness, MBC has been shown to improve depressed client outcomes with medium-effect size improvements over usual care [12, 1, 13]. A meta-analysis indicated that MBC is particularly effective for improving outcomes when depressed clients are not demonstrating progress and by reducing client deterioration [1, 14]. In terms of reach, MBC has greater reach potential than complex EBPs (e.g., CBT) that involve multiple theory-specific components targeted at single disorders. MBC has transtheoretical (relevant for use by clinicians regardless of background) *and* transdiagnostic (effective in enhancing usual care for numerous disorders) relevance [11]. We conceptualize MBC as a minimal intervention needed for change (MINC; [15]) as it may optimize diverse usual care offerings for depression by enhancing the clinician’s ability to target session interventions to clients’ unique symptom profiles and needs without requiring clinicians undergo months of retraining or shed their established theoretical orientation [11].

In terms of utility, MBC aligns with the Patient Protection and Affordable Care Act (P.L. 111–148) by focusing on monitoring outcomes and satisfying meaningful use requirements [16]. MBC presents a systematic approach for selecting and adapting interventions [17] by flagging clients who are not improving and highlighting treatment targets [13]. The National Institute of Mental Health’s (NIMH) nationwide public health clinical trial, the STAR*D (Sequenced Treatment Alternatives to Relieve Depression), demonstrated MBC’s utility for guiding both medication and psychotherapeutic interventions [18]. MBC also has established utility for promoting care coordination across disciplines [19]. Finally, MBC provides the basis for evaluating subsequent EBP implementation efforts through a foundation of outcome monitoring, which can be built into the soon to be ubiquitous electronic health record technologies.

Despite MBC’s demonstrated effectiveness, reach, and utility, recent estimates suggest that fewer than 20 % of PhD level clinicians, master level clinicians, and psychiatrists routinely measure mental health symptoms prior to each session [20–22]. MBC has great potential for implementation success by design, yet barriers such as attitudes and feasibility exist [23], and a gap remains between documented MBC effectiveness and use in practice. Previous attempts to integrate MBC into real world settings have focused on the development of standalone feedback systems [24]. Few studies have investigated the strategies necessary to integrate MBC into community mental health while (1) interfacing MBC with existing system requirements and clearly delineating the necessary implementation strategies and (2) taking into account stakeholder perceptions and needs when building the implementation approach.

### Current study

The long-term goal of this research project is to provide generalizable and practical recommendations about strategies that promote MBC implementation, fidelity, and sustainment in CMHCs. To realize this goal, this study will test a standardized versus a tailored approach to implementing MBC at Centerstone, the largest provider of community-based mental health services in the USA. In this study, MBC will center on administration of the Patient Health Questionnaire-9 Item Version [25]. The PHQ-9 contains nine items that map directly onto the symptoms of a major depressive episode (DSM-IV TR; [26]) (e.g., trouble sleeping, little interest) and one item pertaining to impairment. The PHQ-9 thus provides a depression severity score capturing the previous 2 weeks. The PHQ-9 is one of the best-validated depression measures used in over 1000 research studies [27]. The PHQ-9 has depression severity cutoff scores, is sensitive to change [28], and is useful for weekly administration as an indicator of treatment effectiveness [29]. Three diagnostic meta-analyses and a recent review have confirmed the good sensitivity and specificity of the PHQ-9 in making a major depressive disorder diagnosis [27].

Although touted as superior, tailored implementations have rarely been compared to standardized approaches [30, 31]. Moreover, recent research has demonstrated a need to adapt EBPs to fit the specific context in which they are being implemented, particularly if they are to be sustained [32]. This proposal reflects a movement in the field of implementation science to compare planned EBP adaptations to standardized EBP approaches [2, 33]. This study is a mixed methods implementation-effectiveness hybrid evaluation of a standardized versus tailored approach to MBC implementation on both clinician-level (e.g., MBC fidelity) and client-level (depression symptom change) outcomes. Central to this investigation is the need to identify contextual factors (e.g., attitudes, resources, process, etc.) that mediate MBC fidelity and optimize client outcomes.

#### Primary guiding model

**Fig. 1:**
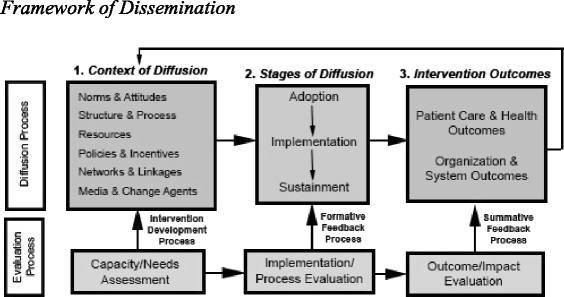
Framework of dissemination

The *Framework for Dissemination* [34] is the study’s guiding conceptual model as it is derived from the best available implementation research and includes a three-phase evaluation process for use with academic-community partnerships (see Fig. 1). The context of diffusion within this model highlights six domains theorized to determine successful implementation (see Fig. 1, Box 1): (1) norms and attitudes, (2) structure and process, (3) resources, (4) policies and incentives, (5) networks and linkages, and (6) media and change agents.

The first three domains affect the willingness and ability of stakeholders to implement and sustain new interventions [34]. Norms and attitudes represent the knowledge, expectations, beliefs and values of stakeholders (e.g., beliefs about a novel practice’s value for an organization). Organizational structure and process reflect the way an organization is structured and operates to deliver services, including sets of characteristics such as organizational mission, size, governance and decision-making processes, and types of services offered. Resources include the varied forms of financial, physical, human, social, and political capital necessary to implement, spread, and sustain new practices. The other three domains relate to sources of information and influence through which potential adopters learn about and assess innovations [34]. Policies and incentives constitute rewards or sanctions embedded in regulatory, funding, and organizational rules and programs that alter the costs and benefits of adopting a new service offering (e.g., financial bonuses, opportunities for enhancing training). Networks and linkages reflect the conduits among organizational and community participants that enable the flow of information and social support around potential adopters (e.g., communication with other sites or provider organizations, staff relationships). Finally, media and change agents represent specifically active sources of information with credibility related to new practices or services (e.g., external trainers, consultants, or internal champions, experts, and cheerleaders).

Taken together, the context of diffusion within the *Framework for Dissemination* organizes the evaluation of key contextual factors that may promote or hinder the implementation and sustainment of EBPs in CMHCs [34]. As such, this model will not only allow for an examination of contextual mediators of implementation success, but it will also be used to guide the tailored approach to implementation. Finally, the model will drive the three-phased evaluation process (see Fig. 1, Row 2): (1) Capacity/Needs Assessment, (2) Implementation/ Process Evaluation, and (3) Outcome/Impact Evaluation.

#### Complementary testable model

This study will also test a recently proposed model, the *Dynamic Sustainability Framework* (DSF; [2]). The *DSF* promotes testing the longstanding hypothesis that program drift from “EBP fidelity” leads to a voltage drop in client outcomes when results are compared to effect sizes observed in efficacy trials. Chambers et al. [2] contend that an alternative hypothesis is important to consider, intentional prospective EBP adaptations informed by stakeholders and that account for relevant multilevel contextual factors may optimize implementation, sustainment, and client outcomes, particularly when adaptations are made iteratively to fit the dynamic context. The present study will test this model by comparing a standardized condition to a tailored condition. The standardized condition requires (via an implementation guideline) that MBC be integrated prior to each and every psychotherapy session according to its documented efficacy (e.g., [1]), whereas the tailored condition will encourage implementation teams (comprised of CMHC clinicians, staff, administrators, and research staff) to adapt the MBC guideline and implementation by taking into account site-specific contextual factors. Consultation in the standardized condition will be offered to clinicians with the goal of achieving and maintaining MBC fidelity, whereas consultation in the tailored condition will support contextual and intervention changes to best fit the particular site. This study will address three specific aims.

*Aim 1*: To compare the effect of standardized versus tailored MBC implementation on clinician-level (*1a*) and client-level (*1b*) outcomes. We hypothesize that tailored implementation will outperform standardized in terms of (*H1a*) MBC fidelity and (*H1b*) reducing client depression severity.

*Aim 2*: To identify contextual mediators of MBC fidelity. We hypothesize that contextual mediators (e.g., structure, norms, etc.) will be leveraged in the tailored condition, but serve as barriers in the standardized condition.

*Aim 3*: To explore the impact of MBC fidelity on client outcomes as a preliminary test of the Dynamic Sustainability Framework [2]. We hypothesize that adapted MBC protocols (tailored condition) will outperform weekly administration of PHQ-9s (standardized condition) with respect to clinically significant change in depression severity from intake to week 12.

## Methods/design

### Study context

Centerstone is the largest provider of community-based mental health services in the USA – delivering psychotherapy, social work services, psychiatric services, and health promotion/disease prevention interventions to over 140,000 children, families, and adults each year. Centerstone has a physical presence in five-states (Tennessee, Indiana, Illinois, Kentucky, and Florida) and hosts the Knowledge Network (a national collective of mental health agencies focused on evidence-based care). Centerstone also maintains the largest repository of community mental health data in the USA—containing data from over 350,000 patients, 20 million service records, and 2 million prescriptions. This “data warehouse” exists to facilitate mental health services research and populates an innovative analytics platform (Enlighten Analytic) to guide clinical decision-making and agency management. The clinical research, program evaluation, informatics, and grant writing arm of Centerstone—Centerstone Research Institute (CRI)—works with the clinical enterprise to prioritize the standardization of care through EBP delivery. Centerstone initiated a partnership with the principal investigator (CCL) in 2011 to develop clinical pathways for depression. A workgroup, comprised of three regional directors from clinics across Indiana and Tennessee and the CRI Director of Clinical Research, convened for monthly meetings over the course of 2 years. MBC was identified as the EBP of focus given its relevance and potential utility for improving client outcomes across diagnoses—a necessary feature given the highly complex and comorbid presentations of Centerstone clientele.

### Conditions

#### Overview of the blended implementation protocol

Both conditions (standardized and tailored) will employ the same blended protocol of implementation strategies (i.e., combination of discrete strategies [35]) to remove time and resource confounds (e.g., ensure differences across conditions are not due to one having more training). The blended protocol of implementation strategies was defined using the guiding model (*Framework for Dissemination* [34]), the best available literature on promoting MBC implementation (e.g., [13, 36–41]), the MBC intervention framework, the partnership goals, and the pilot study results. Table 1 depicts and describes the protocol and unique focus of the implementation strategies across conditions. The order of strategies will proceed as follows within each site’s active implementation period: (1) embed PHQ-9 in electronic health record; (2) conduct needs assessment; (3) offer initial training; (4) set guidelines; (5) form implementation team; (6) conduct triweekly group consultation meetings. Following the active implementation phase, the implementation team will be encouraged to continue to meet to promote sustainment. A similar blended protocol of implementation strategies led to successful MBC implementation using the PHQ-9 with 90 % completion with over 30,000 clients each quarter at Group Health Cooperative, a consumer-governed, non-profit, health care system in Washington and Idaho [40].

**Table 1:** Standardized versus tailored protocol and focus

Contextual factor	Implementation strategies	Standardized focus	Tailored focus
Resources	Paper-based PHQ-9 with score entered in EHR for clinician review	Client completion of PHQ-9 on paper and score entered in EHR for review by the clinician	Client completion of PHQ-9 on paper and score entered in EHR for review by the clinician
Networks and linkages	Form implementation teams for each site consisting of the site administrator, a clinician identified as an opinion leader, a self-nominated MBC champion, an office professional staff member, and research staff using data from the initial needs assessment. Each team will meet triweekly over the course of the active implementation period	Team meetings focus on monitoring and promoting MBC fidelity per the guideline	Team meetings focus on identifying remaining barriers
Policies and incentives	Guideline for PHQ-9 administration frequency	Each session w/client	Determined by site
Norms and attitudes	Initial MBC training	Standardized training material	Tailored training material targeting identified barriers from the needs assessment
Structure and process	Progress note modifications	For clinician score review	For clinician score review
Media and change agents	Triweekly consultation with experts	Consultation focuses on MBC fidelity, particularly on incorporating clinician PHQ-9 score review into sessions, encouraging discussion of scores with clients, and providing tips on targeting lack of progress	Consultation focuses on targeting identified barriers in addition to MBC fidelity. However, emphasis will be placed on tailoring review, discussion, and targeting lack of progress to the site-specific PHQ-9 guidelines to address contextual and other barriers as they are identified throughout the course of implementation

The standardized condition includes all aforementioned strategies in the order listed above and described in Table 1. Tailored implementation refers to the *responsive application of implementation strategies and content matched to determinants of practice* (i.e., barriers) *identified via a needs assessment and formative evaluations*. The same strategies outlined in the standardized condition will be employed in the tailored condition (Table 1); however, the content will be tailored to the context of each site. The needs assessment will employ rapid ethnography methods to reveal contextual factors that may serve as barriers to the implementation process. The results of the needs assessment will be shared with a site’s implementation team, which will convene to adapt and define the site-specific guidelines for PHQ-9 completion and other intervention components. For instance, it may be that Site X decides that monthly PHQ-9 administration is optimal with respect to feasibility and clinical utility. Conversely, Site Y may prefer to have clients complete the PHQ-9 every other session. As described in Table 1, training will be offered to all enrolled clinicians using tailored materials that will target the identified barriers from the needs assessment. For instance, if clinicians at a particular site perceive the PHQ-9 to be irrelevant to clients, training content will incorporate client perspectives on its utility. If clinicians indicate that lack of time is a barrier, then training will incorporate experimentation to streamline review and discussion of scores. Different members of the investigative team will lead training and consultation in the two conditions to ensure differentiation. A contamination interview and analysis will occur at the end of the active implementation to determine what, if any, crossover occurred between conditions.

### Phases and procedures

#### Phase 1: randomized trial, implementation and sustainment

**Fig. 2:**
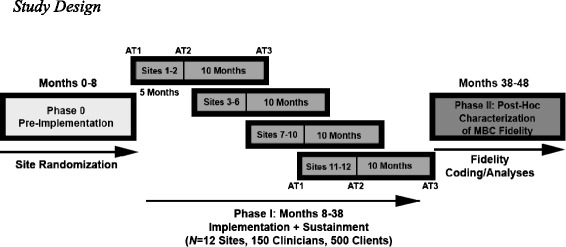
Study design

The dynamic cluster randomized implementation trial will be completed across 30 months and include 12 sites in four cohorts. Each cohort will have 5 months of active implementation. Specifically, matched sites (based on size and urban/rural status) are randomized to either early or later stage implementation in four cohorts (2–4 sites per cohort spaced 5 months apart), with half the sites randomized to the standardized and half to the tailored condition (Fig. 2). The timing of this protocol is based on the published work of Miller et al. [42] as well as the successful naturalistic MBC implementation at Group Health [40]. Table 2 provides an outline of the steps that will occur in Phase 1. All of the listed steps will be repeated across each of the four cohorts.

**Table 2:** Implementation Phases I and II overview

Evaluation aim	Evaluation activities
*Phase 1: randomized trial, implementation and sustainment*	
Needs assessment (AT1; Fig. 1, Box 1)	*Engage in the baseline mixed methods needs assessment*
	(a) A subset of clinicians (*N* = 5–8) identified via purposive sampling representing extreme variation will participate in a 1.5-h focus group
	(i) Rapid Ethnography will then be used to uncover site-specific insights that will guide the content of training and consultation *in the tailored condition only*
	(b) All enrolled clinicians will complete the battery of baseline measures (Table 3)
	*Implementation teams form*
	(a) Opinion leaders (Childers, 1986) and self-nominated MBC champions will be identified from the needs assessment
	*Clinicians Participate in 4-h MBC training workshop*
	*Implementation teams convene triweekly and optional consultation offered to participating clinicians*
	(a) Implementation team meetings and consultation sessions will be audio-recorded and coded (see Additional file 1).
	(b) A site-specific team member will also log meetings (using Additional file 1) and the data will be triangulated to evaluate consistency across sites
Implementation/process evaluation (AT2; Fig. 1, Box 2)	*Engage in mixed methods reassessment*
	(a) Conduct an additional round of focus groups with clinicians
	(b) Re-administer the baseline surveys to clinicians
	*Site implementation teams encouraged to continue to meet to promote MBC sustainment without the research personnel present*
Outcome/impact evaluation (AT3; Fig. 1, Box 3)	*Engage in mixed methods reassessment*
	(a) Conduct an additional round of focus groups with clinicians
	(b) Re-administer the baseline surveys to clinicians
	*Conduct focus groups with implementation team to review their experience and site progress since the research personnel exited the team.*
*Phase 2: post hoc characterization of MBC fidelity*	
Outcome/impact evaluation	*Measure MBC fidelity via data collected in the electronic health record*
	(a) Clinician reported client PHQ-9 scores
	(b) A report to indicate whether the clinician looked at the scores
	(c) Clinician self-report of whether they discussed the PHQ-9 scores with the client
	*Triangulate MBC fidelity outcome with client post-session text message surveys and objective therapy session coding*
	*Focus group data formally coded for mixed method analysis*

*Note*: AT1 = assessment time 1—prior to MBC implementation; AT2 = assessment time 2–5 months after the needs assessment; AT3 = assessment time 3–10 months after implementation/process evaluation or 15 months after the baseline needs assessment

#### Phase 2: post hoc characterization of MBC fidelity

Consistent with the Dynamic Sustainability Framework [26], the approach to MBC implementation may be adapted by sites in the tailored condition. Therefore, characterizing fidelity for the tailored condition will need to reflect the guideline established for each site. The steps of Phase 2 are delineated in Table 2.

### Site selection and randomization

Fifty Centerstone sites were examined for eligibility from Tennessee and Indiana based on criteria such as size (>5 clinicians providing individual psychotherapy to depressed adult clients) and rural versus urban status. Twelve sites were matched using three characteristics: number of clinicians, number of adult depression diagnoses, and urban versus rural status. A dynamic cluster randomization trial was selected to enhance feasibility of capacity management [43]; the design dictated four time cohorts (see Fig. 2). Prior to randomization, the number of sites for each of the four time cohorts was determined as follows: 2, 4, 4, and 2.

Randomization was implemented following Chamberlain et al. [44] and Brown, et al. [45] who employed a similar sequential design. Sites were first randomized into eight site clusters to fill the eight cells in the design (4 time cohorts × 2 conditions). Cluster assignment was optimized through assigning sites to 10,000 random permutations of eight site clusters, four with one and four with two sites. The random assignment with the smallest distance was selected as the optimal set of site cluster (i.e., the randomly created site cluster combination with the greatest similarity in site clusters was selected). Next, site clusters were randomly assigned to one of the four time cohorts and then, within the time cohort, site clusters were randomly assigned to the standardized or tailored condition.

### Participants

#### Clinician inclusion criteria

A total of 187 clinician participants will be recruited across 12 sites (target *N =* 150). The number of sites and clinicians was determined by a simulation-style power analysis to detect a small effect size. Within each enrolled site, clinicians will have the option to participate if they (1) are at least 40 % full-time equivalent, (2) provide individual psychotherapy, (3) treat adults with depression, and (4) conduct sessions in English.

#### Clinician data collection

Table 3 summarizes the clinician battery and assessment time points: Baseline Needs Assessment (AT1, prior to MBC implementation); Implementation/ Process Evaluation (AT2, 5 months in, following the active implementation phase; Additional file 1); and Outcome/ Impact Evaluation (AT3, 15 months in, 10 months after research personnel have ceased active involvement). Each contextual factor of diffusion [28] will be assessed via self-report and qualitatively via focus groups with a subset of clinicians. MBC fidelity is the main clinician-level implementation outcome. Barriers to PHQ-9 completion will also be collected for the first 5 months of implementation via clinician self-report within the electronic health record.

**Table 3:** Quantitative clinician measures: potential contextual mediators

Construct	Description	Time point (month)		
		0	5	15
Demographics	An adapted 16-item version of the one developed by Lewis & Simons (2011) to assess clinician demographic information (e.g., age, gender, ethnicity) as well as training (e.g., degree level, measurement-based care training) and treatment (e.g., theoretical orientation, caseload) information	X		
Norms	A 6-item measure of subjective (3 items) and descriptive (3 items) norms developed based on the guidelines and considerations put forth by theory of planned behavior measurement development manuals (Azjen, 2006; Francis et al., 2004)	X	X	X
Attitudes	*Monitoring and Feedback Attitudes Scale* (MFA; Jensen-Doss, Becker, Smith, Lyon, Lewis, Stanick, & Hawley, *in prep*) is a 17-item measure of clinician’s attitudes about “‘routine’ progress monitoring and providing feedback to clients about treatment progress.” It has demonstrated good internal consistency and consists of three scales: MFA Benefit, MFA Harm, and MFA Trust	X	X	X
	*Evidence-based practices attitudes scale* (EBPAS; Aarons, 2004) is a 15-item measure of clinician’s attitudes toward adopting evidence-based practices, consisting of one total score and four scales: Appeal, Requirements, Openness, and Divergence. It has demonstrated good psychometrics, displaying good internal consistency (Aarons, 2004; Aarons et al., 2007) and construct and convergent validity (e.g., Aarons et al., 2007; Aarons, 2004; Aarons & Sawitzkey, 2006; Aarons, 2006)	X	X	X
Culture & Climate	*Survey of Organizational Functioning* (TCU SOF; Broome et al., 2007) is 162-item measure of an organization’s resources, job attitudes, readiness to change, workplace practices, climate, and training exposure and utilization. The SOF is comprised of 32 scales, which includes 23 scales from the Organizational Readiness for Change (ORC; Lehman et al., 2002) measure and nine additional scales measuring workplace practice and job attitudes, with all of these scales demonstrating acceptable psychometrics (e.g., Broome et al., 2007; Broome et al., 2009; Lehman et al., 2002).	X	X	X
	*Implementation Climate Scale* (ICS; Ehrhart, Aarons, & Farahnak, 2014) is an 18-item measure assessing a clinician’s perception of the strategic climate of the organization, specifically the facets of the organizational climate that are essential for successful implementation of EBPs. It consists of a total score and six factors: Selection for openness, Recognition for EBP, Selection for EBP, Focus on EBP, Educational Support for EBP, and Rewards for EBP. It has displayed sound psychometrics, including good to excellent internal consistency and construct-based evidence of validity (Ehrhart et al., 2014)	X	X	X
Structure and process	*Barriers and Facilitators to Implementing* survey (Salyers, Rollins, McGuire, & Gearhart, 2009) is a 21-item measure of the potential barriers and facilitators to implementing an innovation. It contains a total facilitator score and three subscales: Agency leadership support, Program-level structures, and Job-related structural supports with two open-ended questions: “What facilitated/helped you provide MBC?” and “What were the challenges/barriers to providing MBC?” The measure has demonstrated excellent internal consistency across the three subscales (Salyers et al., 2009)	X	X	X
	*Clinic Systems Project (CSP) Director Survey* (Schoenwald, Kelleher, Hoagwood, Landsverk, & Glisson, 2003) is a structured interview that will be reduced and adapted for relevance to the study and conducted with the clinic directors of each site and the executive director. This survey assesses the infrastructure of the organization and sites, such as staffing, turnover, supervision, services, and previous implementation practices, and financing. The CSP Director Survey interview also includes 21-items from the Dimensions of Organizational Readiness-Revised (DOOR-R; K. Hoagwood et al., 2003), which asks the director to rate the domains or factors (e.g., management support, treatment fit or match with the organization’s mission) s/he perceives to be critical for successful implementation of new treatments and services	X		
Policies and incentives	*Qualitative Focus Groups* will be used to explore the domain of Policies and Incentives (see Additional file 2), as no quantitative measures exist	X	X	X
Resources	*Survey of Organizational Functioning* (TCU SOF; Broome et al., 2007)’s Resources scale is a 25-item scale from the SOF. The scale has five subscales: Offices, Staffing, Training, Computer Access, and e-Communications	X	X	X
Networks and linkages	*Sociometric Questionnaire* (Valente et al., 2007) is a 3-item sociometric questionnaire that will be given to identify professional, personal, and advice relations among staff (Valente, Chou, & Pentz, 2007) and calculate the density and centrality of the network. The three questions will include: a) “Which clinician is a colleague you go to as a source of information on client care-related matters;” b) “Which clinician is a colleague with whom you discuss you clinical work;” and c) “Which clinician is a colleague with whom you are friends?” The questionnaire will be paired with an alphabetized roster of all of the clinicians at each site, in which the respondent designates his or her relationship with each individual. An adjacency matrix will be used to record information about the relationships or ties between each pair of clinicians (Wasserman & Faust, 1994). This information will then be entered into social network analysis software , which will allow for measurement of network metrics, in particular centrality and density	X	X	X
Media and change agents	*Opinion Leadership Scale* (OLS; Childers, 1986) is a 6-item opinion leader self-identification scale that will be employed to identify clinician opinion leaders who may serve as influential change agents. The six items were adapted for relevance to clinical work and rated by clinicians. It has demonstrated good internal consistency and validity (Childers, 1986)	X	X	X
	*Qualitative Focus Groups* will be used to collect information on internal and external media influence since no quantitative measures currently exits to examine these constructs	X	X	X

#### Client recruitment

A total of 625 depressed adult clients (target *N =* 500) will be recruited for enrollment in this study; approximately three clients will be recruited per participating clinician. As this is a pragmatic trial [46], client participation criteria is very inclusive: (1) age 21 and above; (2) depression is one of the primary treatment foci based on clinician diagnosis (e.g., major depressive disorder, dysthymic disorder, depressive disorder NOS, adjustment disorder with depressed mood); (3) significant depressive symptom severity (PHQ-9 total score >9 reflecting moderate depression); (4) receipt of individual psychotherapy; (5) fluency in English; (f) new client beginning treatment; and (6) client is receiving services from an enrolled study clinician during the proposed funding period. Exclusion criteria are minimal, an inability to sign the consent due to lack of competence or inability to read.

CRI staff will identify eligible clients via nightly electronic health record queries. Clients will be contacted by telephone and administered the PHQ-9 [25] to confirm eligibility prior to their first therapy session. Only clients scoring greater than 9 (reflecting at least moderate depression) on the PHQ-9 will be invited to participate. Once a client has been enrolled, the study team will flag the client in the electronic health record to initiate study procedures for the clinician.

#### Client data collection

The goal of client data collection is to determine if the measurement schedules and use of MBC by clinicians in the standardized versus tailored conditions have differential effects on depression symptom outcomes. Clients will be invited by participating clinicians to complete the PHQ-9 in the waiting room according to site-generated guidelines (prior to each session in the standardized condition, site-specific schedule in the tailored condition). Client PHQ-9 responses will be entered into the electronic health record by clinicians to enable review of scores over time (via a graphical interface) and to facilitate discussion of symptom trajectories in session. Given that this is an implementation trial, there is potential for variance in the completion/administration of the PHQ-9 instrument. To enhance data quality, enrolled clients in both conditions will complete the PHQ-9 at baseline and at week 12 of treatment in a phone call with study staff. The 12-week window reflects a commonly used time period in randomized clinical trials during which depressive symptoms are expected to remit. We will also supplement active research data collection with regularly collected administrative data (e.g., multiaxial psychiatric diagnoses, employment status, medication, etc.) captured in intake and progress review reports in the electronic health record. Finally, to substantiate the clinician self-report of PHQ-9 integration into the session, the client will be asked to respond to five-item text message surveys immediately following each session inquiring about clinician MBC fidelity.

### Aim 1:

#### To compare the effect of standardized versus tailored MBC implementation on clinician-level (1a) and client-level (1b) outcomes

We hypothesize that tailored implementation will outperform standardized in terms of (*H1a*) MBC fidelity and (*H1b*) reducing client depression severity.

### Quantitative measures

#### MBC fidelity

Data pertaining to MBC fidelity will be collected via three mechanisms: (1) clinician report in the electronic health record; (2) client report via text messaging; and (3) objective coding of a subsample of clinician-client sessions. The trichotomous fidelity outcome variable will be computed using the following data from the electronic health record as follows: (a) clinician recorded client PHQ-9 scores (score 0 = No PHQ-9; 1 = PHQ-9); (b) a report to indicate whether the clinician looked at the scores (score 0 = did not review PHQ-9; 1 = reviewed PHQ-9); and (c) clinician self-report of whether they discussed the PHQ-9 scores with the client (score 0 = did not discuss; 1 = discussed with client in session). Overall MBC fidelity scores can range from 0 to 3 reflective of “0 = none”, “1 = mild”, “2 = moderate”, and “3 = excellent” fidelity. This fidelity outcome variable will be triangulated with client post-session text message surveys and objective session coding as the three reports (clinician, client, and objective coder) may not align [47, 48].

#### Client depressive symptom severity: patient health questionnaire 9-item version (PHQ-9)

In addition to the PHQ-9 serving as the central focus of the MBC (symptom monitoring), client scores on the PHQ-9 [25] at baseline and week 12 serve as the primary effectiveness client-level outcomes.

### Statistical analyses

Multilevel generalized linear models will be used to assess hypotheses for standardized versus tailored MBC implementation for both models of clinician and client outcomes. The multilevel generalized linear model framework includes continuous outcomes and extends the linear model to accommodate nonlinear outcomes by including a distributional assumption and link function (e.g., a binomial distribution with a logistic link function will be used to implement logistic models). Specifically, we will examine the effect of the implementation condition on MBC fidelity (ordered categorical outcome; range 0–3 reflecting client completion, clinician review, discussion in session) measured across time. Multilevel models account for the non-independence of repeated measurements within participants and non-independence due to sites [49]. Models for clinician and client outcomes will be constructed in an identical manner by following a model building-sequence recommended by Singer and Willett [50] in which (1) empirical growth plots will be examined, (2) an unconditional means model will be fit, (3) an unconditional linear growth model will be fit, (4) unconditional nonlinear growth models (a quadratic model) will be fit, (5) unconditional linear and nonlinear growth models will be compared using the Akaike Information Criterion to identify the best model of change across time, and (6) level-2 and level-3 predictors will be added.

#### Power calculations

A power analysis for the Aim 1 multilevel models was conducted using Monte Carlo studies in which power is the proportion of significant effects (2-tailed α = 0.05) for a parameter of interest across all simulated data sets fit with the same model [51, 52] using MPlus (version 7). For each model, 10,000 data sets were simulated and analyzed. Data in the Monte Carlo studies were simulated with the goal of identifying the smallest detectable effect size with power ≥ 0.80. Dropout was simulated to reflect an increase of 5 % missing data per wave. Repeated measurements were nested within participants (ICC = 0.50) and participants were nested in sites (ICC = 0.05). Average effect sizes for Aim 1 analyses were computed using an approximation of Cohen’s *d* for growth models [53]. Using this metric, the study is sufficiently powered to detect effect sizes as small as *d* = 0.46 for the clinician models (*N* = 150) and effect sizes as small as *d* = 0.30 for the client models (*N* = 500).

### Aim 2:

#### To identify contextual mediators of MBC fidelity

We hypothesize that contextual mediators (structure, norms, etc.) will be leveraged in the tailored condition but serve as barriers in the standardized condition.

### Quantitative measures

The dependent variable for Aim 2 is the implementation outcome of MBC fidelity as described in Aim 1. The independent variables summarized in Table 3 include the six factors delineated in the context of diffusion (norms and attitudes; structure and process; resources; policies and incentives; networks and linkages; media and change agents) from the *Framework of Diffusion* as potential mediators, assessed at baseline (AT1), 5 months into the implementation process (AT2), and 15 months into the implementation process (AT3). See Table 3 for a complete listing of the quantitative measures.

### Focus groups

Focus groups will be conducted with 5–8 clinicians at each site at baseline (AT1) after the active implementation phase (AT2) and at 10 months post active implementation phase (AT3). Focus group participants will be selected in collaboration with the clinic director using purposive sampling for extreme variation [54]. Focus groups will serve to identify key barriers to MBC implementation, trace the ways in which they affect or mediate processes of care and client outcomes, and evaluate whether these barriers change over the course of active implementation and sustainment of MBC. We will develop a focus group script in collaboration with an expert in qualitative inquiry in order to maximize information gleaned regarding the *Framework for Dissemination* domains highlighted above (Additional file 2). Equal number of questions will be presented to focus group participants to evaluate norms and attitudes, structure and process, policies and incentives, resources, networks and linkages, and media and change agents. Equivalent numbers of follow up questions (e.g., 1–2) will also be included in the focus group script to allow for adequate coverage of potential barriers. The focus group protocols will be standardized across sites in order to capture comparable information about barriers to MBC implementation.

### Statistical analyses

#### Quantitative analyses

We will examine mediation models in which contextual factors (based on clinician-completed surveys, Table 3; and, clinician selection of reasons for MBC deviations captured in the electronic health record) mediate the impact of the implementation condition on both clinician- and client-level outcomes. We will assess differences in MBC fidelity between conditions by examining clinician, client, and organizational factors using multilevel generalized linear models that will provide a general framework for assessing group differences for a variety of outcome distributions (normal, binomial, Poisson, etc.) that we anticipate will be necessary to characterize the factors impacting MBC fidelity. Mediation models will be two-level models (i.e., clinicians nested within sites) or three-level models (clients nested within clinician nested within sites) in which outcomes are measured at the individual level, condition is assigned at the site level, and mediators are at the clinician or site level. For assessing mediation in a multilevel context, models will be constructed following recommendations from Preacher, Zyphur, and Zhang [55]. To assess mediation, we will test whether (1) implementation condition predicts the change in the mediator (path *a*), (2) the mediator predicts growth change in MBC use (path *b*), (3) condition predicts change in the outcome (path *c*), and (4) whether the implementation condition’s effect on MBC use becomes significantly weaker when controlling for the mediator (path *c’*) [56, 57]. We will also apply recommendations from Kraemer, Wilson, Fairburn, and Agras [58] to demonstrate that change in the mediator precedes change in MBC use. The indirect effect (i.e., the product of paths *a* and *b*) will be tested using biased-corrected bootstrapped confidence intervals [59].

#### Qualitative analyses

Rapid ethnography [60] will be used to synthesize the needs assessment data in the tailored condition to characterize participant experiences and to provide efficient data analysis for the purpose of tailoring the MBC training approach. Focus groups across conditions and across time points will also be analyzed separately to characterize participant responses within each site using a formal coding procedure. We will identify and code analyzable units of meaning in the focus group transcripts using multiple trained coders to enhance reliability. An iterative approach to coding will resolve disagreements through research team discussion. Codes will also be assigned based on contextual factors using the *Framework of Dissemination* [34]. Inductive analyses based on emergent themes rooted in grounded theory will be conducted. The final list of consensus codes will include themes established *a priori* and through emergent themes analysis.

#### Mixed methods analyses

Mixed methods will be used to integrate findings from Aim 2 using a QUAN + QUAL structure (wherein both types of data are collected simultaneously) to achieve the function of data expansion for the purposes of evaluation and elaboration [48]. Using the US NIH guidelines for mixed methods best practices [61], we will connect the quantitative and qualitative datasets in QSR N-Vivo to allow for case-specific pattern identification and hypothesis testing. First, we will enter quantitative MBC fidelity data into N-Vivo to categorize sites (within each condition) into four fidelity groups (categorized as “none”, “low”, “moderate”, “high”) in order to reveal patterns among the contextual factors that appear to influence level of MBC fidelity. This approach will allow us to distinguish factors that might explain the differences in the quantitative findings, and notably MBC fidelity. Second, given that both the focus group questions and the quantitative measures will align with the *Framework for Dissemination* [34], we will be able to elaborate on the quantitative findings that require further explanation through the process of expansion using qualitative results.

#### Power calculations

For Aim 2, we conducted power analyses for the hypothesized mediation effects with the same assumptions described above but with the use of effective samples size estimates (an ICC corrected sample size) based on a design effect adjustment [62] in order to use standard effect size metrics. The *κ*^2^ effect size for mediation (0.01, 0.09, and 0.25 represent small, medium and large *κ*^2^, respectively) was computed [63]. We are sufficiently powered to detect effect sizes as small as *κ*^2^ = 0.16 for the clinician models and effect sizes as small as *κ*^2^ = 0.10 for the client models.

### Aim 3:

#### To explore the impact of MBC fidelity on client outcomes thereby testing the DSF [2]

We hypothesize (*H3*) that adapted MBC protocols (tailored condition) will outperform weekly administration of PHQ-9s (standardized condition) with respect to clinically significant change in depression severity from intake to week 12.

### Statistical analysis

We will assess the impact of MBC fidelity (standardized versus tailored) on clinically significant change observed in each client between intake and session 12 using generalized linear mixed models. Models will represent the three-level structure (i.e., clients are nested within therapists and therapists are nested within sites) with a binary outcome, representing whether a client exhibited clinically significant change using the reliable change criterion [64], modeled with a binomial distribution with a logistic link function.

#### Power calculations

For Aim 3 analyses, we are sufficiently powered to detect effect sizes as small as *r* = 0.27 for clinicians and as small as *r* = 0.21 for clients. The planned sample sizes are consistently sufficient for detecting medium-effect sizes for clinician outcomes and small effect sizes for client outcomes, with the power to treat site as a random effect based on six sites per condition [65].

### Trial status

The Indiana University Institutional Review Board has approved all study procedures. The Pre-Implementation phase (Phase 0) of the study was completed in May 2015, and Phase 1 subject recruitment and data collection began in June 2015 with the first cohort of clinic sites.

## Discussion

### Innovation

This study is innovative in at least three ways. First, our focus on testing strategies to implement MBC in community mental health is innovative because this simple MBC framework may be the minimal intervention needed for significantly reducing the burden of depression on society [11]. For the treatment of depression, we opted to focus on MBC rather than a complex, theoretically driven EBP like Cognitive Behavioral Therapy not only because of the MBC implementation gap, but also because the simplicity and accessibility of the MBC framework will likely reduce the number of implementation barriers. Moreover, MBC has been isolated as a core component of many EBPs. Therefore, identifying effective implementation strategies for MBC would build the case for a phased or staged approach to full package EBP implementation to determine whether later EBP implementations enhance outcomes beyond improvements observed with MBC.

Second, we will modify the electronic health record system to provide clinically relevant data to providers who use MBC, such as symptom trajectory graphs, alerts when suicidality is endorsed, etc. With electronic health record prevalence increasing, this approach presents a generalizable and cost-effective method for engaging in systematic outcome monitoring that maximizes therapeutic benefit and aligns with meaningful use requirements [66].

Third, the majority of existing implementation research has focused either on descriptive studies that explore barriers and facilitators (determinants of practice) or on comparisons of *a priori* selected implementation strategies that generally neglect contextual tailoring of the interventions. A qualitative analysis of 22 implementation studies revealed that few focused on matching strategies to determinants of practice [67]. A critical research agenda has emerged seeking to identify, “how and why implementation processes are effective” [68] by experimentally evaluating implementations that are tailored to the context. Recent findings support the need to adapt evidence-based practices during the implementation process [69, 70], but no studies, to our knowledge, have directly compared this approach to standardized EBP implementation.

### Limitations

In addition to these and other strengths, this approach to implementation of MBC has limitations. First, this implementation effort focuses on outcomes for standardized and tailored MBC as implemented within multiple sites of a single behavioral health organization. While it is anticipated that many of the strategies necessary to facilitate MBC implementation will align with previous research efforts, the degree of generalizability of the standardized and tailored approaches to other sites and settings is unknown. Second, clinician and client turnover across participating clinic sites may limit the sample size obtained, despite researcher efforts to exceed recruitment targets. Fortunately, literature suggests that participatory approaches to organizational change in practice patterns result in lower clinician attrition [71]. Third, the tailored approach to implementation is site-specific by design and does not allow for the evaluation of the effectiveness of specific tailored implementation protocols. Instead, the study approach seeks to evaluate the effect of tailoring more broadly. However, the mixed methods component of the study will explore the variety of MBC tailoring approaches and protocols utilized by the sites in this study that can be more closely tested in future research to determine which may be most effective with respect to implementation and client outcomes.

### Expected impact

Measurement-based care aligns with priorities delineated in the Patient Protection and Affordable Care Act (P.L. 111–148), notably systematic evaluation of services [16] and meaningful use requirements of electronic health records; however, systematic monitoring of client/consumer outcomes remains a challenge for providers of community-based mental health services. This study has the potential to impact public health through identification of practical and generalizable approaches to implementing MBC into CMHCs. Moreover, the evaluation of contextual mediators across 12 diverse Centerstone sites may reveal common barriers to be directly addressed in future research. Evidence-based intervention frameworks, such as MBC, are rarely the focus of implementation efforts. Thus, uncovering unique or previously identified contextual factors impacting implementation is critical to advancing implementation science. Finally, exploring MBC fidelity and its relation to client outcomes may reveal a “minimal intervention needed for change” (MINC; [15]) and the mechanisms through which MBC has an effect.

## Additional files

Additional file 1:
**MBC Barriers Log.** (DOCX 58 kb) Additional file 2:
**Focus Group Guide.** (DOCX 115 kb)

## Abbreviations

CBT: cognitive behavioral therapy
CMHC: Community Mental Health Center
CSP: Clinic Systems Project
CRI: Centerstone Research Institute
DOOR-R: Dimensions of Organizational Readiness-Revised
DSF: Dynamic Sustainability Framework
EHR: Electronic Health Record
EBP: Evidence-Based Practice
EBPAS: Evidence-Based Practice Attitudes Scale
MBC: Measurement-Based Care
MINC: Minimal Intervention Needed for Change
MFA: Monitoring & Feedback Attitudes Scale
NIH: National Institute of Health
OLS: Opinion Leadership Scale
ORC: Organizational Readiness for Change
PHQ-9: Patient Health Questionnaire – 9
SOF: Survey of Organizational Functioning
