# Supplementary material for: Implementing measurement-based care (iMBC) for depression in community mental health: a dynamic cluster randomized trial study protocol
Source: Implement Sci. 2015 Sep 7;10:127. doi: 10.1186/s13012-015-0313-2 (PMC4561429; doi:10.1186/s13012-015-0313-2)
Supplement: Additional file 1: — MBC Barriers Log. (DOCX 58 kb) [file 13012_2015_313_MOESM1_ESM.docx]

| **Contextual Barrier** | **Present? (Yes/No)** | **Specific Issue** | **Issue Resolution Plan** |
| --- | --- | --- | --- |
|  |  |  | **(Action, Timeline, Outcome)** |
| Norms & Attitudes | | | |
| "A range of attitudes and knowledge about particular health conditions, expectations and priorities toward types of treatments or client populations and collectively held beliefs and values. " | | | |
|  |  |  |  |
|  |  |  |  |
|  |  |  |  |
|  |  |  |  |
|  |  |  |  |
|  |  |  |  |
| Structure & Process | | | |
| Refers to the structure and way an organization operates (e.g., mission, size, decision-making process, services offered) | | | |
|  |  |  |  |
|  |  |  |  |
|  |  |  |  |
|  |  |  |  |
|  |  |  |  |
|  |  |  |  |
| Resources | | | |
| Varied forms of financial, human, social, and political capital (e.g., funding, staff with expertise, infrastructure [information technology, office space) | | | |
|  |  |  |  |
|  |  |  |  |
|  |  |  |  |
|  |  |  |  |
|  |  |  |  |
|  |  |  |  |
| Policies & Incentives | | | |
| The incentives (or disincentives) embedded in regulatory practices, funding & reimbursement programs, and rules and policies. Incentives may be monetary or non-financial (e.g., intrinsic rewards or burden of learning new skills, more or less pleasant work environments, and individual or organizational reputation) | | | |
|  |  |  |  |
|  |  |  |  |
|  |  |  |  |
|  |  |  |  |
|  |  |  |  |
|  |  |  |  |
| Networks & Linkages | | | |
| The linkages and connections among organizations and other stakeholders that enable social support and flows of information within a community or healthcare system. | | | |
|  |  |  |  |
|  |  |  |  |
|  |  |  |  |
|  |  |  |  |
|  |  |  |  |
|  |  |  |  |
| Media & Change Agents | | | |
| External sources of information and influence on innovative practices. Examples include media outlets (e.g., print or electronic) and "change agents" (e.g., consultants, professional associations, public health officials, therapeutic companies, and other marketers of health service interventions, and academics. | | | |
|  |  |  |  |
|  |  |  |  |
|  |  |  |  |
|  |  |  |  |
|  |  |  |  |
|  |  |  |  |
