# Supplementary material for: Implementing measurement-based care (iMBC) for depression in community mental health: a dynamic cluster randomized trial study protocol
Source: Implement Sci. 2015 Sep 7;10:127. doi: 10.1186/s13012-015-0313-2 (PMC4561429; doi:10.1186/s13012-015-0313-2)
Supplement: Additional file 2: — Focus Group Guide. (DOCX 115 kb) [file 13012_2015_313_MOESM2_ESM.docx]

**Additional File 2**

**Focus Group Guide**

Based on the Context of Diffusion in the *Framework of Dissemination* (Mendel et al., 2008)

| **Domain** | **Indicator** | **Question 1** | **Alternative** | **Follow up** |
| --- | --- | --- | --- | --- |
| **Norms and Attitudes** | Descriptive norms: Perception of what colleagues in the agency are doing with respect to identified provider behaviors | What is the "norm" here with respect to measurement-based care (i.e. routinely measuring client outcomes prior to session to inform care)? | Tell me about the role of routine outcome monitoring here at your site. | Do you notice other people in your organization using MBC? How so? |
|  | Injunctive norms: Perception of what an individual thinks that colleagues believe they should be doing with respect to identified provider behaviors | What do you suppose your colleagues think about the role of measurement-based care in your clinic? | How do your colleagues feel about measurement-based care? | Do you feel social pressure from others to use MBC? How so? |
|  | Attitudes: | How do you feel about new practices like MBC? | How important or useful is MBC? | If positive, what do you like about it? |
|  |  |  |  | If negative, what don't you like about it? |
|  | | | | |
| **Structure and Process** | This set of contextual factors relates to the structure and way an organization operates, including differences in mission, size, decision-making process, and services offered. | Tell me about the impact the organization's operations has on implementing new practices like MBC. | What effect do operations, mission, size, decision-making processes, and services delivered have on implementing a new practice like MBC? | Follow up on each that was not addressed previously: operations, mission, size, decision-making processes, and services delivered |
|  | | | | |
| **Policies and Incentives** | Incentives (or disincentives) embedded in regulatory policies, funding and reimbursement programs, and rules and policies of adopting organizations themselves that alter the costs and benefits supporting new behaviors | Tell me about how policies within your organization facilitates or inhibits implementing new practices like MBC | How do you think that policies (e.g., regulations, funding and reimbursement programs, and rules) affect the implementation of a new practice like MBC? | Follow up on each that was not addressed previously: regulatory policies, funding and reimbursement programs, and rules and policies |
|  |  | Tell me about how incentives/disincentives in your clinic facilitates or inhibits implementing new practices like MBC | How do you think that incentives/disincentives affect the implementation of a new practice like MBC? | Can you give me examples of what incentives work? |
|  |  |  |  | Can you give me examples of what disincentives work? |
|  | | | | |
| **Resources** | Financial, human, social, and political capital | Tell me about how financial support facilitates or inhibits implementing new practices like MBC? | How much does financial support impact your ability to implement new practices like MBC with clients? | How so? |
|  |  | Tell me about how support from others (e.g. supervision/tech support) facilitates or inhibits implementing new practices like MBC. | How much does support from others impact your decision to implement new practices like MBC with clients? | How so? |
|  |  | Tell me about how political capital facilitates or inhibits implementing new practices like MBC. | How much political capital do you feel you have for implementing new practices like MBC? | How so? |
|  | | | | |
| **Networks and Linkages** | Linkages and connections among organizations and other stakeholders that enable social support and flows of information within a community or healthcare system | Tell me about how staff discussions facilitate or inhibit implementing new practices like MBC. | How do staff discussion help or hurt using new practices like MBC? | Can you give me an example of what these discussions typically look like? |
|  |  | Tell me about how staff provide social support to other staff in the organization to facilitate or hinder implementing new practices like MBC. | How does staff social support help or hinder using new practices like MBC? | Can you give me an example of what social support looks like? |
|  |  | Tell me about how other stakeholder (managers, directors) discussions facilitate or inhibit implementing new practices like MBC. | How does other stakeholder discussion help or hurt using new practices like MBC? | Can you give me an example of what these discussions typically look like? |
|  |  | Tell me about how other stakeholders (managers, directors) provide social support to other staff to facilitates or inhibits implementing new practices like MBC. | How does other stakeholder discussion social support help or hinder using new practices like MBC? | Can you give me an example of what social support looks like? |
|  | | | | |
| **Media and Change Agents** | External sources of information and influence on innovative practices. Media outlets—print or electronic | Tell me about the importance of receiving training from external trainers (not within the organization) for facilitating new practices like MBC | How do staff at this clinic typically respond to external trainings? | When this occurs, what kind of training is necessary to support new practices like MBC? |
|  |  | Tell me about how external organizations or representatives providing information about new practices facilitates or hinders implementing new practices like MBC. | How do staff at this organization typically respond to this type of information? | When this occurs, what type of information is provided that is most helpful to implementing new practices like MBC? |
